# Towards real-time myocardial infarction diagnosis: a convergence of machine learning and ion-exchange membrane technologies leveraging miRNA signatures†

**DOI:** 10.1039/d4lc00640b

**Published:** 2024-10-04

**Authors:** Xiang Ren, Ruyu Zhou, George Ronan, S. Gulberk Ozcebe, Jiaying Ji, Satyajyoti Senapati, Keith L. March, Eileen Handberg, David Anderson, Carl J. Pepine, Hsueh-Chia Chang, Fang Liu, Pinar Zorlutuna

**Affiliations:** a Department of Aerospace and Mechanical Engineering, University of Notre Dame Notre Dame IN 46556 USA pinar.zorlutuna.1@nd.edu +1 (574)631 8543; b Department of Applied and Computational Mathematics and Statistics, University of Notre Dame Notre Dame IN 46556 USA; c Department of Chemical and Biomolecular Engineering, University of Notre Dame Notre Dame IN 46556 USA; d Division of Cardiology, Department of Medicine in the College of Medicine, University of Florida Gainesville FL 32611 USA

## Abstract

Rapid diagnosis of acute myocardial infarction (AMI) is crucial for optimal patient management. Accurate diagnosis and time of onset of an acute event can influence treatment plans, such as percutaneous coronary intervention (PCI). PCI is most beneficial within 3 hours of AMI onset. MicroRNAs (miRNAs) are promising biomarkers, with potential of early AMI diagnosis, since they are released before cell death and subsequent release of larger molecules [*e.g.*, cardiac troponins (cTn)], and have greater sensitivity and stability in plasma *versus* cTn regardless of timing of AMI onset. However, miRNA-based AMI diagnosis can result in false positives due to miRNA content overlap between AMI and stable coronary artery disease (CAD). Accordingly, we explored the possibility of using a miRNA profile, rather than a single miRNA, to distinguish between CAD and AMI, as well as different stages following AMI onset. First we screened a library of 800 miRNA using plasma samples from 4 patient cohorts; no known CAD, CAD, ST-segment elevation myocardial infarction (STEMI) and STEMI followed by PCI, using Nanostring miRNA profiling technology. From this screening, based on machine learning SCAD and Lasso algorithms, we identified 9 biomarkers (miR-200b, miR-543, miR-331, miR-3605, miR-301a, miR-18a, miR-423, miR-142, and miR-132) that were differentially expressed in CAD, STEMI and STEMI-PCI and explored them to identify a miRNA profile for rapid and accurate AMI diagnosis. These 9 miRNAs were selected as the most frequently identified targets by SCAD and Lasso, as indicated in the “drum-plot” model in the machine learning approach. We used age-matched patient samples to validate selected 9 miRNA biomarkers using a multiplexed ion-exchange membrane-based miRNA sensor platform, which measures specific miRNAs, and cTn as a control, simultaneously as a point-of-care device. Findings from this study will inform timely and accurate diagnosis of AMI and its stages, which are essential for effective management and optimal patient outcomes.

## Abstract 2

Rapid diagnosis of acute myocardial infarction (AMI) is crucial for optimal patient management.

## Graphical abstract

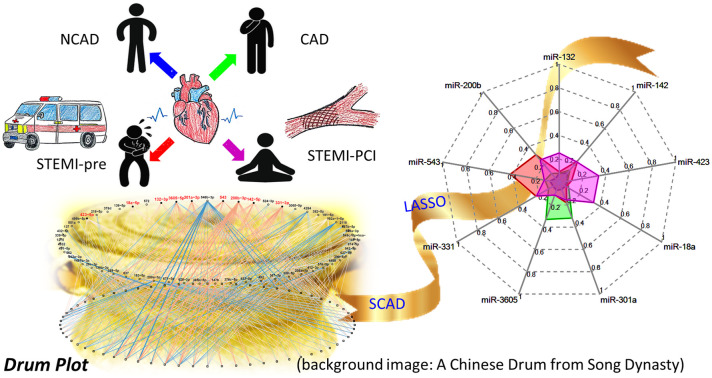

## Introduction

Coronary artery disease (CAD) is the leading cause of death in the US. A common presenting symptom is chest pain, often resulting in acute myocardial infarction (AMI)^1^ that requires timely percutaneous coronary intervention (PCI) as the preferred clinical treatment.^2–5^ In its chronic form, on the other hand, CAD may contribute to heart muscle dysfunction over time and can lead to serious complications, including heart failure.^6^ Therefore, the diagnosis of CAD and the assessment of potential for an acute event are critical in clinical management. Currently the diagnoses of CAD and AMI are primarily based on the clinical presentation (*i.e.*, chest pain), the electrocardiogram (ECG),^7,8^ contrast-enhanced coronary computed tomography (CCTA),^9^ and laboratory measurement of circulating biomarkers (*i.e.*, cardiac troponins (cTn) and creatine kinase-MB (CKMB)) from plasma samples.^10–12^ These biomarkers for AMI have low positive predictive reliability and are not entirely specific for coronary events, as there are non-cardiac pathologies that cause elevated troponin levels such as chronic kidney injury and/or sepsis.^13–16^ Furthermore, cTns are inherently late markers of AMI since they are released from cells upon membrane rapture. In addition, while the ECG can be obtained within minutes of presentation, cTn results typically take several hours after blood sampling. This delay in diagnosis can lead to more cardiac muscle loss, undue interventions, and decrease the effectiveness of treatment.^17–20^ Given these considerations, diagnosing AMI and deciding on PCI treatment are a critical time-dependent issue. There is a need for biomarkers of myocardial damage with higher sensitivity and specificity, as well as rapid presentation and detection, for timely management of AMI.

MicroRNAs (miRNAs) are small (17–22 nucleotides), non-coding RNAs that regulate gene expression post-transcriptionally^21^ found in various body fluids, including whole blood and plasma. They have recently emerged as promising tools involved in many pathophysiological conditions including cardiovascular diseases as they are produced by cells deliberately under pathophysiological conditions as a first-response as opposed to cTns that emerge because of cell death. Hence, miRNAs have been explored as early detection biomarkers for various diseases. Studies have reported that miRNAs are dysregulated in CAD and circulating miRNA profiles can serve as potential biomarkers for prognosis and rapid and accurate diagnosis of AMI and CAD.^22,23^ As miRNA turnover is much faster than proteins, information gathered from miRNAs can be used for early detection and severity assessment of the acute phase without need for additional testing. However, since the AMI often times develops as a result of chronic CAD, miRNA-based AMI diagnosis can result in false positives due to miRNA overlap between AMI and CAD. As such, current miRNA-based approaches have limitations in distinguishing between chronic stable CAD and AMI and have been deemed so far as clinically not reliable.

Most studies exploring miRNA expression in CAD patients use miRNAs isolated from cell-free plasma for convenience and availability of the samples.^24,25^ A qualitative study of human and rat serum and plasma samples highlights the preference for using plasma, especially in translational miRNA studies, due to higher aligned reads in plasma than in serum.^26^ In contrast, another study comparing miRNA content of plasma and whole blood samples reported that only a few miRNAs were differentially expressed in both sources, while most of the information was lost in plasma samples.^27^ Therefore, it is important to consider the source of miRNAs and the method used for the detection of the miRNA, especially plasma-derived exosomal miRNA, as the source and the methods can influence the results obtained. Recently, the Nanostring nCounter platform® has been introduced for the detection, quantification, and assessment of miRNA expression. This platform offers the advantage of direct screening of more than 800 miRNAs in patient samples without potential biases that might arise from RNA amplification, however, it is expensive and the sample preparation process for Nanostring requires lysing the exosome, which can cause miRNA loss due to additional freeze–thaw cycles. Additionally, traditional data analysis methods fall short to process the large datasets generated by Nanostring, especially when screening more than 800 miRNAs, which translates into over 800 dimensions. While both principal component analysis (PCA) and partial least-squares discriminant analysis (PLS-DA) can reduce dimensions in a limited space, they are not suitable for analyzing over 800 parameters with a limited sample size (*n* = 6 for each category). Therefore, we implemented smoothly clipped absolute deviation (SCAD) and least absolute shrinkage and selection operator (Lasso) methods to analyze bulky Nanostring miRNA expression data of the 4 different cohorts (NCAD, CAD, STEMI-pre and STEMI-PCI) have here.

Mainly due to the low efficiency of extraction and increased error margin from extensive sample preparation and processing miRNAs are at low in concentration. In addition, miRNA isolation from exosomes traditionally involves chemical lysing, causing further potential miRNA degradation or loss. Moreover, traditional protein (*i.e.*, cTn) sensing methods use western blots or enzyme linked immunosorbent assay (ELISA) and have high uncertainties in sample preparations, antibody processing, and testing protocols. We previously demonstrated that our multiplexed ion-exchange membrane-based miRNA (MIX.miR) sensor platform can accurately measure miRNA concentrations at physiologically relevant picomolar (pM) levels.^28^ Increased detection sensitivity enabled MIX.miR to overcome the main limitation of current methods (*i.e.*, RT-qPCR), and we identified miRNAs in plasma that had been previously reported for only whole blood samples from patients. Having used this membrane for other proteins before,^29^ we adapted it for cTn detection as an internal control. The MIX.miR has three main advantages over traditional methods, increased detection sensitivity, integrated chemical-free exosome lysing unit using surface acoustic waves (SAW), and integrated ion-exchange membrane (IEM) at a relatively low cost. While traditional biological methods of testing miRNAs require extensive work with sample preparation and miRNA duplication, resulting in miRNA loss and long assay time detrimental to this application, the MIX.miR sensor platform can accurately detect miRNAs with a concentration level of 1 pM within one hour. Additionally, traditional lysing involves mechanical or chemical interventions on the exosomes, which often interfere with miRNAs and potentially cause miRNA degradation or loss. With the SAW lysing unit relying on physical forces, MIX.miR breaks the exosomes and extracellular vesicles without introducing chemical impurities.^28,30,31^ Furthermore, the sensor constructed with IEM can easily be functionalized into protein sensors for untreated blood.^29,32,33^ The IEM-based sensor can utilize the principle of ELISA at a much lower cost with better detection sensitivity.^29^

In the current study, we used the MIX.miR platform to determine the differential expression of miRNAs in patients with CAD, STEMI, and reference patients without CAD. We then analyzed the data with SCAD and Lasso methods to assess the predictive value of miRNAs for CAD and STEMI. We hypothesized that miRNA profiles would represent the stage of STEMI and evaluate the efficacy of PCI better than the current reference standards. Point-of-care miRNA and high-sensitivity cTn testing platforms have potential to be within emergency medical services, such as ambulances, to facilitate efficient and rapid diagnosis of AMI at the pre-hospital stage.

## Materials and methods

### Clinical samples

Clinical samples were provided by UF Health in Gainesville. The protocols are described in our previous work^28^ and briefly described in the ESI.† The modeling groups for Nanostring nCounter miRNA dataset are recorded as “random samples”; while the testing groups with matched ages, diabetes, and AMI onset hours are recorded as “matched samples”.

### Oligoprobes and calibration miRNAs

Oligoprobes and calibration miRNAs were purchased from Integrated DNA Technologies, Inc. The well-known miRNA biomarkers for MI diagnosis are miR-1, miR-208b, and miR-499. New miRNA biomarkers studied in this work are miR-200b, miR-543, miR-331, miR-3605, miR-301a, miR-18a, miR-423, miR-142, and miR-132. The detailed information of the oligoprobes is listed in the ESI.† All oligoprobes and calibration miRNAs were aliquoted and stored at −20 °C. The human cardiac troponin T antibody pair (ab270345, Abcam, Waltham, MA) is aliquoted and stored at 4 °C until usage. The “detection” antibody is for the silica beads, while the “capture” antibody is for the functionalization of the AEM sensors.

### miRNA selection by machine learning and regularized statistical modeling

The Nanostring dataset comprise expression levels of 809 miRNAs from 4 groups of subjects: CAD patients (*n* = 6), pre-treatment MI patients (*n* = 6), AMI patients after PCI treatment (*n* = 6), and non-CAD healthy controls (*n* = 6). Regularized logistic regression was employed to select biomarkers that might relate to different patient groups. For the logistic regression, we compare the following pairs of groups: (1) NCAD *vs.* CAD, (2) NCAD *vs.* MI-pre, (3) NCAD *vs.* MI-PCI, (4) CAD *vs.* MI-pre, (5) CAD *vs.* MI-PCI, (6) MI-pre *vs.* MI-PCI; in addition, we compared (7) NCAD + CAD *vs.* MI-pre (8) NCAD + CAD *vs.* MI-PCI (9) NCAD *vs.* MI-pre + MI-PCI, (10) CAD *vs.* MI-pre + MI-PCI, (11) NCAD + CAD *vs.* MI-pre + MI-PCI, leading to a total of 11 logistic regression models. In each model, the first group is used as a reference group, meaning the models model the odds of being in the second group *vs.* in the first group. A positive coefficient associated with a biomarker from a model means that an increase in the biomarker is associated with a higher odd of being in the second group.

We employed the LASSO (least absolute shrinkage and selector operation)^34^ and the SCAD (smoothly clipped absolute deviation),^35^ to regularize the variable selection (technical details on LASSO and SCAD are provided on the ESI†). We implemented the leave one out cross validation (LOOCV) to tune the penalty parameter for LASSO and SCAD. In addition to running the above logistic regression models with LASSO and SCAD penalties on the full dataset with 809 miRNAs, we also run the models on three subsets of the 809 biomarkers after manual screening based on domain knowledge. The first subset contains purely cardiac specific miRNAs (450); the second subset contains cardiac specific and vasculature miRNAs (565); the third group contains cardiac specific, vasculature, cerebral vasculature, and rare cardiac disease miRNAs (576). Among 11 logistic regression models, no biomarker was selected in 3 regression models regardless of the penalty term or the tuning parameters, which are NCAD *vs.* MI-Pre, CAD *vs.* MI-PCI, and MI-Pre *vs.* MI-PCI. Taken together, the mRNA selection results were obtained from 8 regression models with LASSO or SCAD penalty, respectively, among the full set of biomarkers and 3 subsets, leading to a total of 64 regressions models. Nine frequently selected miRNAs biomarkers among the 64 regression models, taken together with domain knowledge, were chosen to move onto the next stage, which is validation in the lab.

The analysis was performed in R. For LASSO regression, R package “glmnet v4.1.4” was used; for SCAD, R package “ncvreg v3.13.0” was employed.

### Technical details on LASSO and SCAD regularization

The loss function in logistic regression is the negative log-likelihood function
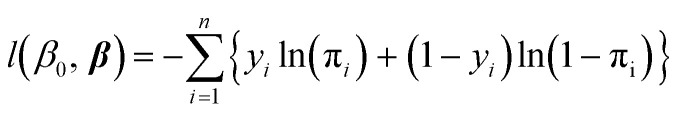
where
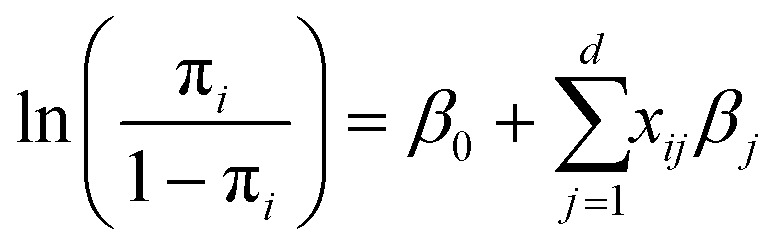
for *i* = 1, 2, …, *n*, ***x***_*i*_ is a *p* × 1 vector that contains the values of *p* miRNA biomarkers in sample *i* and *y*_*i*_ ∈ {0, 1} is a binary group indicator. *β*_0_ is the intercept and ***β*** = (*β*_1_, *β*_2_, … *β*_*p*_) is a *p* × 1 vector of regression coefficients. The loss function of the penalized logistic regression, compared to the regular logistic regression, has an additional penalty term,
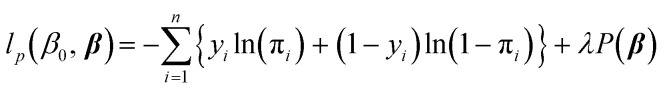
where the penalty term *λP*(***β***) consists of tuning parameter *λ* and the penalty function *P*(***β***). Penalized regression is often used for variable selection and regularizing parameter estimation and predictions when *p* is large, especially when *n* is small, so as to improve the robustness and generalizability of the learned model.

The LASSO (least absolute shrinkage and selector operation) and the SCAD (smoothly clipped absolute deviation) are popular choices of *P*(***β***). Specifically,
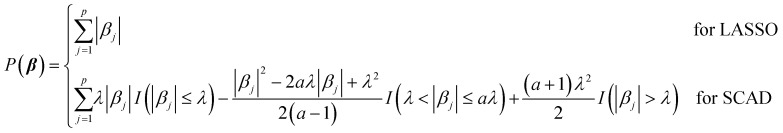
LASSO is likely the most recognized and popular regularizer among practitioners but known to yield biased estimate for non-zero *β*_*j*_. SCAD penalty enjoys the advantage over LASSO to give unbiased parameter estimates (oral estimates) when the parameters are large and is one of the earliest and most influential regularizer to achieve bias reduction compared to LASSO. LASSO is convex regularizer whereas SCAD is non-convex.

The tuning parameter *λ* controls the model complexity and the tradeoff between bias and variance in parameter estimation and prediction. When *λ* = 0, *l*_*p*_(*β*_0_, ***β***; *X*, *Y*) reduces to *l*(*β*_0_, ***β***; *X*, *Y*), so are the parameter estimates. When *λ* → ∞, all ***β*** estimates would be 0 (no variable is selected). When *λ* is in between 0 and *∞*, some ***β*** estimates will be 0 and some are not (the larger *λ*, the more ***β*** estimates will be 0). The miRNAs with non-zero ***β*** estimates are “selected” and deemed relevant in the prediction of *Y*. In practice, the value of *λ* is commonly chosen by a cross validation (CV) procedure – *λ* that yields the minimum mean CV error or the CV error within one standard error of the minimum. We implemented the leave one out CV (LOOCV) procedure due to the small sample sizes.

## Experimental

The discovery of novel biomarkers for CAD, STEMI and following PCI were performed in two steps. First, we tested the different categories by full genetic screening using Nanostring. We picked the significant biomarkers by SCAD and Lasso algorithms. Second, we validated the biomarkers using additional patient plasma samples within specific categories.

The functionalization of miRNAs on MIX.miR sensors is described in our previous work.^28^ Briefly, the MIX.miR sensor was fabricated using the standard replica molding of silicone rubber and polyurethane (PU) molding. The ssDNA of specific miRNA is added onto the AEM after 3,3′,4,4′-benzophenonetetracarboxylic acid (BPDA) and EDAC carboxylation procedures. The antibody for cTnT is added onto the AEM after 0.4 M EDAC and 0.4 M sulfo-NHS (EDAC/NHS) treatment. The silica beads are 50 nm in diameter with detection antibody after EDAC/NHS treatment. To achieve better attachment of the antibody, beads were centrifuged, sonicated (Elmasonic S30h, Elma Schmidbauer GmbH, Singen, Germany) and re-suspend multiple times.^32^

The current–voltage curve (CVC) of the sensors can reveal the concentrations of the targeted biomarkers, either the specific miRNA or the cTnT. Due to the ion-depleting action of the membrane on the side of the functionalized oligonucleotide, the conductivity near that surface membrane is 3 orders of magnitude lower than the bulk or within the charged membrane. The surface layer hence controls the voltage drop and the additional surface charge of the hybridized duplex can sensitively gate the ion current, producing a voltage signal much larger than those from electrochemical sensors. The CVC voltage shifts were correlated with miRNA concentration through calibration curves.

**Fig. 1:**
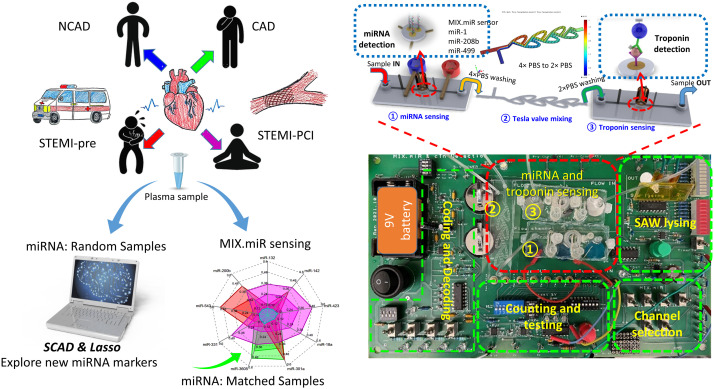
Illustration of the integrated measurement of miRNAs.

The detection board can detect the miRNA and the cTnT at the same time. The sample was introduced from the inlet after SAW lysing.^30^ The exosomal miRNAs were released to the plasma and flowed through the channel with the MIX.miR sensor. The preconcentration unit was balanced with the flow and miRNAs were kept around the MIX.miR sensing area, enhancing the chance of specific miRNAs attaching to the ssDNA. The remaining sample passed through the channel and tubing toward the cTnT sensor on the secondary device. The sample with cTnT was incubated in the cTnT sensing device for 20 min, followed by another 20 min incubation with silica beads with detection antibody. After additional high (4× PBS) and low ionic (2× PBS) wash, the CVC of miRNA and cTnT were obtained separately. We added a microfluidic mixing unit, a customized Tesla valve, between the miRNA sensing device and the cTnT sensing device, such that it has one inlet of 4× PBS and another inlet of DI water with the same flow rate. The 4× PBS was diluted into 2× PBS at the outlet of the Tesla valve, as illustrated in the COMSOL® simulation in Fig. 1. The cTnT in the plasma sample was detected *via* the antibody bound to silica beads in the protein sensing unit (Fig. 1(3)).^30^ Once measured, the voltage shift was used to calculate the miRNA concentration detected in the plasma sample.

## Results

### miRNA screening of the random samples by Nanostring and validation of the miRNAs with MIX.miR sensor

**Fig. 2:**
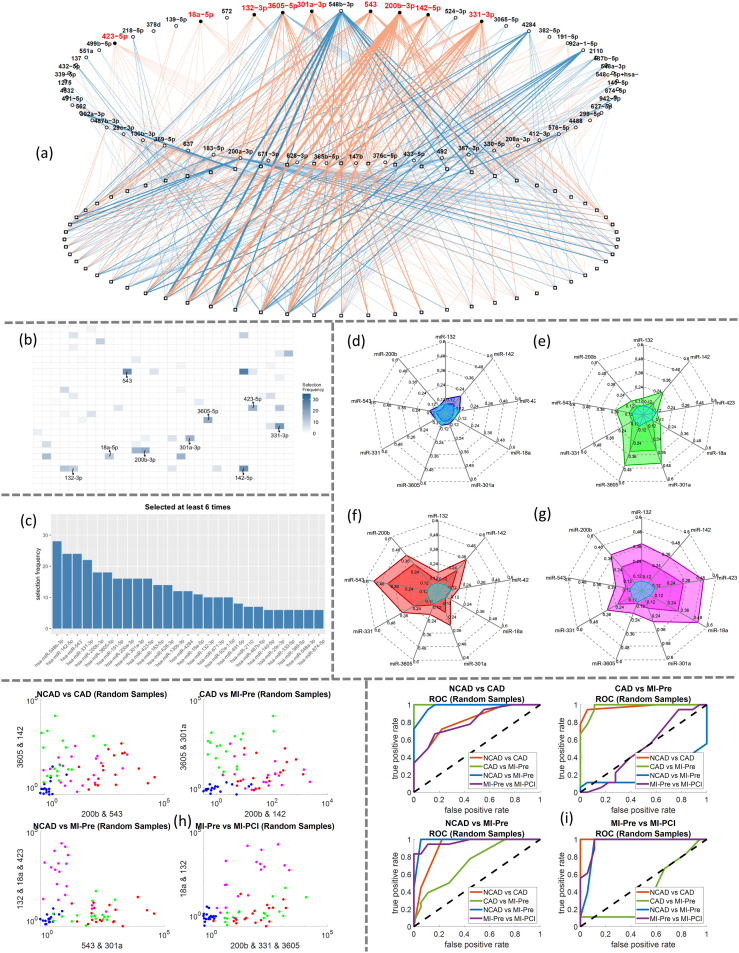
(a) Drum plot on miRNA biomarkers selected from penalized regression models. Each node at the bottom of the drum represents a model and each node at the top of the drum represents a miRNA biomarker selected at least once by the models. A connected line between a top node and a bottom represents the former is selected by the latter. An orange-colored line represents a positive coefficient, and a blue-colored line represents a negative coefficient in the regression. The thicker the line is and the more intense its color is, the larger the magnitude of a coefficient is. The solid nodes at the top of the drum with the red large-font biomarker names are the biomarkers validated subsequently in lab experiments. These biomarkers are selected at least 10 times and most, if not all, are associated with positive coefficients. (b) miRNA biomarker selection frequency heat-map by penalized regression. The mRNAs that moved onto the next stage of lab validation are annotated. (c) Selection frequency of the miRNA biomarker was selected at least 6 times by penalized regression. The mRNAs that moved onto the next stage of lab validation are annotated are those associated with the bar graphs. (d–g) The miRNA detection voltage shifts by MIX.miR sensors on random samples of NCAD (blue), CAD (green), STEMI-pre (red), and STEMI-PCI (magenta), respectively. The inner colored line is the average value; while the outer colored line is the max value; the light blue lines in the center labels the limit of detection (LOD). The LOD is calculated from the calibration curves of each miRNA with linear fittings (Fig. 5). (h) The scatter plot of NCAD, CAD, STEMI-pre and STEMI-PCI random samples with different clustering parameters and objectives; (i) the ROC of different clusters based on the scatter plots with different parameters in (h).

The Nanostring screening recognized 809 miRNAs from the plasma samples. The miRNA data were analyzed by SCAD and Lasso (Fig. 2). SCAD and Lasso are two of the most commonly used methods for variable selection in high-dimensional data analysis, known for their theoretical guarantees in variable selection and practical effectiveness. A “drum plot” was designed to display the results of the biomarker selection from the 64 regression models (Fig. 2a). The selection heat map of the 809 miRNAs from 64 regularized logistic regression models (Fig. 2b), and the selection frequency of the miRNAs that are selected at least 6 times (Fig. 2c) were presented. After factoring in domain knowledge, the miRNAs were finally selected for the next laboratory validation stage as hsa-miR-200b-3p; hsa-miR-543; hsa-miR-331-3p; hsa-miR-3605-5p; hsa-miR-301a-3p; hsa-miR-18a-5p; hsa-miR-423-5p; hsa-miR-142-5p; hsa-miR-132-3p. Note that most of these biomarkers, if not all, are associated with positive coefficients from the regression models.

The association between the selected miRNA and the patient condition such as NCAD, CAD, STEMI-pre, and STEMI-PCI, was further validated with the voltage shifts by MIX.miR sensors (Fig. 2d–g). The studies in miRNA selection by SCAD and Lasso suggested that multiple miRNAs are not completely independent. Fig. 2h presented four different selections of the miRNA combinations in differentiation NCAD, CAD, STEMI-pre, and STEMI-PCI. Based on the scatter plots, we generated the ROC of each combination. The ROC indicated that the 9 miRNA combinations has promising outcomes in differentiating the four groups, which almost reaches 1 in ROC. Some of the ROCs have curves with AUC < 0.5 because not all of the miRNA markers are significant in differentiating among the four groups. However, if consider the 9 miRNA combinations as a spectrum, we are able to differentiate a specific group from NCAD, CAD, STEMI-pre, and STEMI-PCI. In order to validate the selected miRNAs and the robustness, we selected the Matched Samples with age between 60–70 years old, without diabetes, and AMI onset hours below 5–6 hours (for STEMI patient samples) and repeated the detection with MIX.miR sensors.

### Investigation of the robustness of miRNA and cTn

**Fig. 3:**
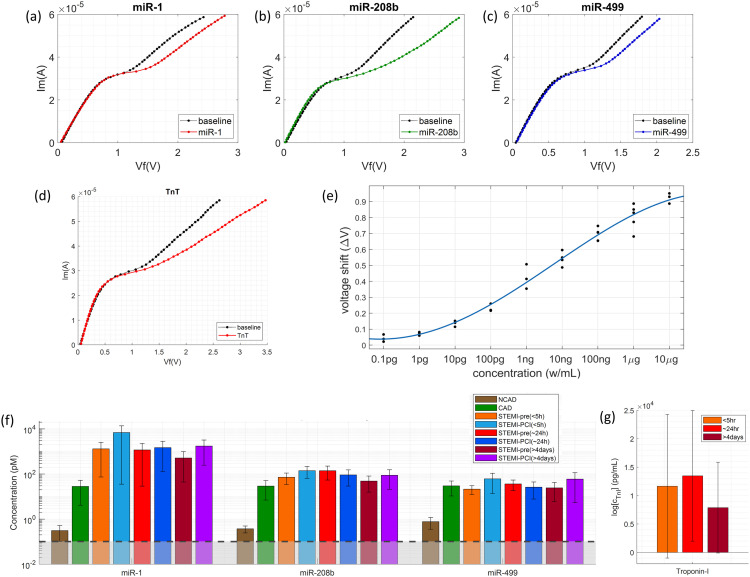
(a–d) The CVC measurement voltage shift of miR-1, miR-208b, miR-499, and troponin T, respectively. (e) The calibration curve of troponin T between voltage shift and concentration. (f) The miRNA concentrations of STEMI samples with different onset hours. The LOD is marked with a dashed line. (g) The troponin I levels of STEMI samples with different onset hours.

We investigated three candidate miRNAs closely associated with STEMI (miR-1, miR-208b, and miR-499) and the biomarker protein in the clinical plasma samples using MIX.miR, the combined miRNA and protein sensing platform (Fig. 3a–e). The molecular concentration feature of MIX.miR reduced the assay time to less than 30 minutes and increased the detection sensitivity by bringing all targets close to the sensors. Note that miRNA levels were comparable across the STEMI patients regardless of the different onset times (<5 h, ∼24 h, and >4 days) from acute events to PCI (Fig. 3f). Both STEMI-pre and STEMI-PCI samples showed high miR-1 levels over two orders of magnitude above normal values, and control patients (NCAD) showed low levels. miR-208b and miR-499 also followed a similar trend to a lesser extent. The miR-1, miR-208b, and miR-499 levels were independent of the intervention status (STEMI *vs.* STEMI-PCI) and the STEMI onset times. We observed that miRNA results were more precise and reliable, as troponin levels were more spread out and greatly affected by the onset times (Fig. 3g).

### Investigation of the selected miRNAs in matched samples by MIX.miR sensor

Here, we used the MIX.miR sensor functionalized with specific single-stranded (ss)DNA to detect the specific cardiac-associated miRNAs in the plasma samples. The 9 potential biomarkers were measured separately by 3 MIX.miR sensors with 3 miRNAs on each.

**Fig. 4:**
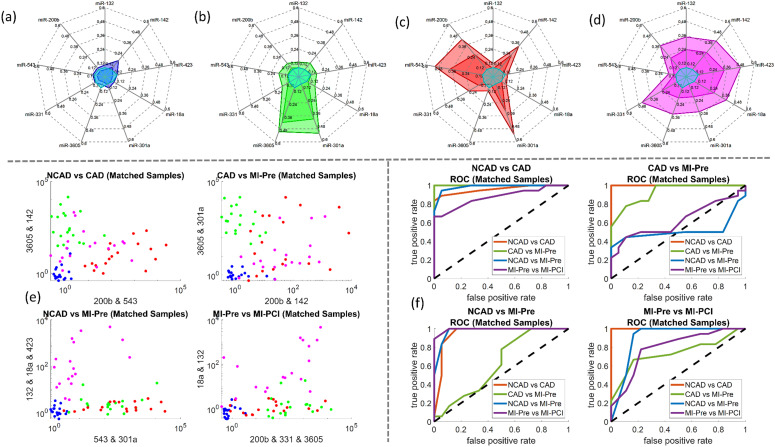
The miRNA detection voltage shifts by MIX.miR sensors on age matched patient testing samples of (a) NCAD (blue), (b) CAD (green), (c) STEMI-pre (red), and (d) STEMI-PCI (magenta), respectively. The inner colored line is the average value; while the outer colored line is the max value; the light blue lines in the center labels the limit of detection. (e) The scatter plot of NCAD, CAD, STEMI-pre and STEMI-PCI testing groups with different clustering parameters and objectives; (f) the ROC of different clusters based on the scatter plots with different parameters in (e).

**Fig. 5:**
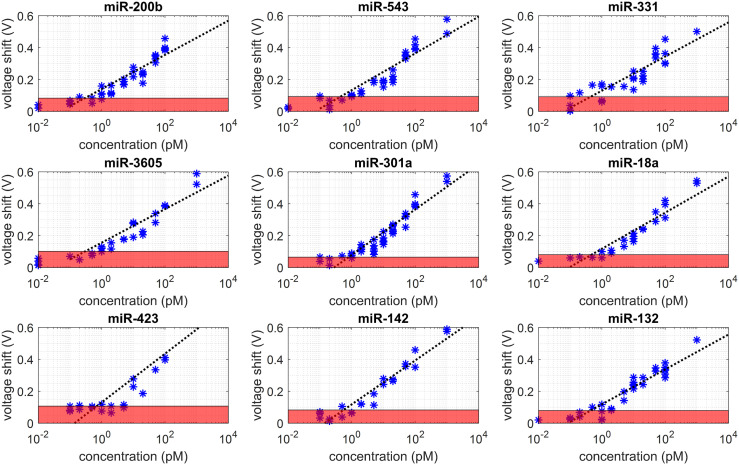
Calibration curves of new miRNA markers and the concentration results of random samples and matched samples of NCAD, CAD, STEMI-pre, and STEMI-PCI. The red regions are the LOD for each miRNA biomarker and also indicated in the radar maps in Fig. 3d–g and 4a–d.

**Fig. 6:**
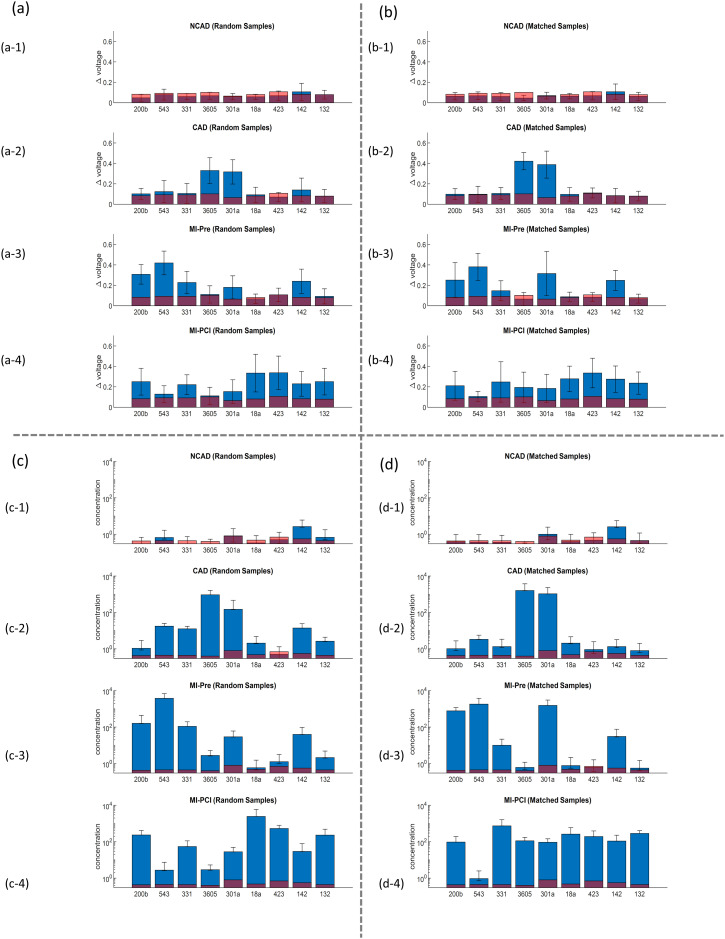
Voltage shifts (a (a-1) to (a-4) and b (b-1) to (b-4)) and converted concentration values (c (c-1) to (c-4) and d (d-1) to (d-4)) of the selected 9 miRNAs from random samples and matched samples of NCAD, CAD, STEMI-pre, and STEMI-PCI.

To exclude the effect of diabetes on STEMI and CAD patients, the testing groups were selected as 55–70 years old patients without diabetes. Additionally, as the best time window of PCI is within 3–5 h of AMI onset, the STEMI patients were selected with the onset time within 5 hours. The MIX.miR sensor measurement results are presented in Fig. 4 and 6. Fig. 5 illustrates the calibration curves of the 9 miRNA biomarkers. The red colored lines are the detection of limit for each miRNA. The CVC voltage shifts were correlated with miRNA concentration through calibration curves. The correlations in the linear region of the Langmuir isotherm are described by
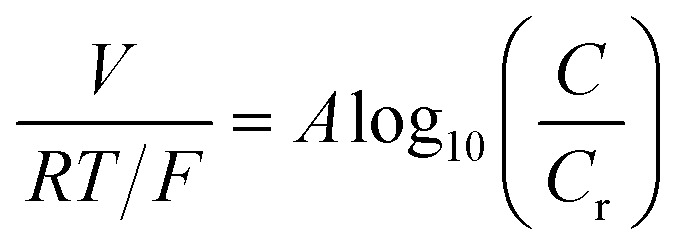
with *A* and reference concentration *C*_r_ being (4.1745, 0.0493), (4.4899, 0.0702), (4.1706, 0.0614), (4.0927, 0.0326), (5.5452, 0.2778), (4.4159, 0.0922), (5.9386, 0.1390), (5.3700, 0.1403), and (4.2641, 0.0836) for the miR-200b, miR-543, miR-331, miR-3605, miR-301a, miR-18a, miR-423, miR-142, and miR-132, respectively. The coefficient *A* is close to the theoretical value of 2 ln(10). The constants are Faraday's constant *F* = 9.648 × 10^4^ C mol^−1^; Boltzmann constant *R* = 8.314 J mol^−1^ K^−1^; and room temperature *T* ≈ 25 °C = 298 K.

Fig. 6 summarizes the 9 miRNA concentrations in both random samples and matched samples. The dark read labels the limit of detection (LOD) in each miRNA. The LOD indicates the limit of the MIX.miR sensor in detecting different specific sequences of the miRNA biomarkers. The LODs are calculated from the calibration curves in Fig. 5. According to the Langmuir isotherm above, the LOD of each miRNA shows minor differences.

The MIX.miR sensor platform captured different miRNA profiles of NCAD, CAD, STEMI-pre and STEMI-PCI samples, supported by the current literature on the selected miRNAs as biomarkers. The CAD patients had high levels of miR-3605 and miR-301a (Fig. 4b), which is in agreement with a previous study that reported differentially expressed miR3605-3p and miR301a-3p in whole blood samples of CAD patients with recurrent MI events.^27^ STEMI-pre patients had high levels of miR-543, 301a and moderate levels of miR-331, 142, and 200b (Fig. 4c). miR-543, miR-301a-3p and miR-200b have been previously associated with CAD presentations including AMI.^36–38^ Additionally, miR-331 was reported to be a significant STEMI biomarker detected before myocardial necrosis markers (*i.e.*, cTnI, miR-208 and -499)^39^ and miR-142-3p elevation was suggested as a potential biomarker for detection and diagnosis of STEMI.^40^ The elevated miR-543 and 301a in STEMI samples dropped after PCI, miR-543 levels even reached the control NCAD levels (Fig. 4d). miR-543 downregulation was reported to mitigate inflammatory response and MI-induced cardiomyocyte apoptosis *via* SIRT-1,^41–43^ reflecting beneficial effects of PCI. The miR-142 and 200b levels in STEMI patients were sustained in STEMI-PCI while miR-423, 18a, 331 and 132 levels increased moderately. miR-18a was previously reported in AMI, where its downregulation promoted autophagy of cardiac cells *via* inactivating Akt/mTOR axis.^44^ Elevated miR-18a in STEMI-PCI samples might indicate suppressed cell apoptosis and potentially improved matrix remodeling, inflammation inhibition and as previously reported in other model systems (*i.e.*, osteoarthritis).^45^ It was reported that plasma levels of miR-423-5p in AMI patients increased prior to PCI and then returned to normal within 6 h after PCI.^46^ Additionally, contrasting our STEMI-PCI results, miR-142-3p was previously found to be decreased in plasma of STEMI patients undergoing PCI with no-reflow.^47^ Such differences in miRNA profile were expected due to the fast miRNA turnover and varying sample timing. Overall, it is clear that miRNA profile rather than a single miRNA level is more informative and MIX.miR can rapidly and accurately measure any miRNA combination corresponding to the disease condition of interest.

## Discussion

There has been growing interest in finding novel miRNA biomarkers for diseases. Advances in large-scale genetic screening technology and rapid growth of bioinformatics studies have led to identification of a range of diagnostic biomarkers including proteins, RNAs, long non-coding RNA (lncRNA), circular RNA (cirRNA), and miRNA for certain diseases. Despite the growing number of publications and novel biomarkers in the past decade, the reliability, repeatability, and significance of these novel biomarkers have not reached the anticipated levels. Currently, the reference-standard diagnosis relies on protein biomarker (cTn) evaluation, but novel miRNA biomarkers haven't made it into clinical practices yet. There are three main reasons for this limitation. First, despite the ability of full genetic profiling technologies to detect large quantities of RNA or miRNA, the resulting data are not fully reliable due to the heterogeneity in patient samples. Factors such as age, medical conditions (*i.e.*, diabetes with insulin treatment or other medications), smoking habits, and medication use affect the biomarker profiles.^48,49^ Second, screening technologies are not fully reliable. Despite advances in genetic analysis equipment, issues with detection accuracy persist, resulting in low specificity (high rate of false positives, and false negatives), especially for miRNAs.^50^ The main reason for this is that the majority of miRNAs of interest are exosomal, and techniques used to extract them (*i.e.*, exosome lysis and miRNA extraction) cause significant loss of miRNA.^51^ Moreover, co-isolation of non-exosomal impurities, low reproducibility, low yield, potential damage of extracellular vesicle families and low throughput of the samples are causing the detection uncertainties of miRNAs. Either the unexpected error in duplication of RNA or incomplete lysis of exosomes may cause measurement error in large-scale genetic screening. In addition, the single-base mutation may cause unexpected error in current RNA measurement technologies. Third, data analysis methods are limited, which is a major problem in bioinformatics. Currently, there is no universally accepted or definitive machine learning or artificial intelligence algorithm that can be applied to all bioinformatics analysis. Appropriate machine learning methods and algorithms are required for analyzing large genetic data to avoid over-defining, especially when the patient sample data set is limited.

Cardiac troponin (cTnT or cTnI) assays have been recognized for improving diagnostic accuracy in early detection of AMI. The normal ranges for cTnT is 0.02–0.13 μg L^−1^, and >0.2 μg L^−1^ is considered an indication of MI. Elevated cTnT levels can be detected as early as 2–4 h after onset of AMI symptoms, and peaking at 12–48 h, and remaining elevated for 4–10 days.^10^ Dynamic changes of cTnT provide information about the onset time and severity of MI, the effectiveness of PCI, and the possibility of reperfusion injury. Despite these benefits, early studies showed that the slow increase and late peak of cTnT levels can lead to missed diagnoses of early-evolving AMI.^52,53^ Therefore, cardiac troponin elevation without a secondary diagnostic measure (CT imaging and ECG) is insufficient for complete diagnosis.^54^ Here, we showed that miRNA measurement together with cTnT provides a multi-dimensional approach for MI diagnosis. The integration of miRNA and troponin testing board provides an ability of rapid “point-of-care” MI diagnosis.

Our measurement of cTnT utilizes the principle of ELISA. The capture antibody is attached on the AEM, and the detection antibody is attached on the silica beads. Commercially available ELISA kits are build-in 48- or 96-well plates with pre-coated antibody. However, our AEM sensor-based cTnT measurement can tune the quantity of testing ports with as many sensors as needed for the patient samples. In addition, the turn-over time of our measurement (*t* = 40 min) is much less than the time in ELISA (*t* = 3 h).

A major benefit of miRNA profiling is that cTnT are released into plasma following cardiomyocyte injury, while miRNAs are released into the plasma at earlier time points by exosomes. Studies have showed that miRNA reached the peak expression 3–12 h earlier than conventional biomarkers (*i.e.*, cTnI, CKMB) in the early phases of AMI,^55^ thus being more sensitive. Here we tested the 9 cardiac-associated miRNAs, miR-200b, miR-543, miR-331, miR-3605, miR-301a, miR-18a, miR-423, miR-142, and miR-132 for STEMI and CAD samples. As demonstrated in the radar map and concentration results, miR-301a is elevated in CAD and STEMI, where miR-301a in STEMI might inherited from CAD conditions. miR-543 is uniquely elevated in STEMI-pre, and miR-200b, miR-331, miR-423, and miR-18a together with a gradual increase indicate the reperfusion injury or STEMI-PCI. To identify CAD from STEMI cases, the results indicate that miR-3605 and miR-301a are joint markers for CAD together when they are co-expressed. From the radar map by patients from Nanostring and MIX.miR sensor results, we are able to identify the cases between NCAD, CAD, STEMI-pre, and STEMI-PCI. Additional miRNA biomarkers can be applied on MI diagnosis and PCI efficacy evaluation, if needed. The problem we deal with has a small sample size (*n* = 24) and a large number of predictor variables (*p* = 809) is a typical small *n* and large *p* problem, SCAD and Lasso are well suited for feature selection and initial screening for this type of problem, compared to some other machine-learning methods that may require large datasets to train a complex model with good performance (*e.g.* deep-learning methods) and that are not suitable for interpretable variable selection problems. Based on SCAD and Lasso analyses of over 800 miRNAs, additional miRNAs could be included, but due to the workload, we only presented 9 miRNAs here. There are chances that some other miRNAs are also useful in diagnosis of STEMI status, such as the ∼20 additional miRNAs in by SCAD and LASSO selections. The SCAD and Lasso are well-regarded in the medical and biological literature for identifying potential biomarkers and have become standard tools in these fields.

The integrated board with miRNA and troponin measurement abilities can be further miniaturized, systemized, and commercialized as a POC device that can be applied in the ambulance for AMI emergency care as a rapid pre-hospital, diagnostic tool. The results can be used to evaluate the severity of AMI and the onset time for deciding the need of PCI.

## Conclusions

Our high sensitivity miRNA sensing with the ability of troponin testing can be applied for emergency care of AMI diagnosis in “future medicine”. Based on the miRNA profiling results, we used SCAD and LASSO to select significant miRNAs from the screening and validated the miRNA results by our MIX.miR sensor platform. Our results indicated that the selected 9 miRNAs are useful in categorizing NCAD, CAD, STEMI-pre, and STEMI-PCI samples. The ability to distinguish between CAD and STEMI would be “a game changer” in patient diagnosis and effective life-saving emergency response. The workflow presents an efficient way to prioritize specific miRNA biomarkers for emerging diseases diagnosis in a timely manner. The clinical approaches for using troponin tests and imaging diagnosis can be significantly improved by this type of integrated board, with both miRNA and troponin rapid assays, which has the potential of further integration and miniaturization for emergency care usage.

## Data availability

All data obtained will be shared with the public openly *via* publications in the rigorous, visible and appropriate scientific journals. Data will be published as soon as it is reasonable. Supplemental data for each published paper will be made available on publisher's website, or Dr. Pinar Zorlutuna lab webpage: https://tissueeng.nd.edu/.

## Author contributions

P. Z. and H. C. initiated the ideas and research goals; X. R. conducted experiments and data collection; G. R., S. G. O., and J. J. assisted experiments and data collection; R. Z. and F. L. performed machine learning related data analysis; X. R., H. C., and S. S. also contributed in partial data analysis; X. R. designed, assembled, and tested the testing PCB; H. C., S. S., P. Z., and X. R. designed and manufactured the multiple MIX·miR sensors; X. R., P. Z., and F. L. selected and picked the targeted miRNA biomarkers; K. M., E. H., C. J. P., and D. A. collected and prepared patient samples, including NCAD, CAD, STEMI-pre and STEMI-PCI; S. S., and H. C. provided useful suggestions and assisted the experimental procedures; X. R. and S. G. O. prepared the manuscript text; S. S., H. C., C. J. P., E. H., and P. Z. edited the manuscript; P. Z. and H. C. provided guidance over the research work.

## Conflicts of interest

There are no conflicts to declare.

## Supplementary Material

LC-024-D4LC00640B-s001

## References

[cit1] C. f. D. C. a. Prevention , Heart Disease Facts, https://www.cdc.gov/heart-disease/data-research/facts-stats/?CDC_AAref_Val=https://www.cdc.gov/heartdisease/facts.htm, (accessed 7/4, 2024)

[cit2] Steg P. G., James S. K., Atar D., Badano L. P., Lundqvist C. B., Borger M. A., Di Mario C., Dickstein K., Ducrocq G. (2012). Eur. Heart J..

[cit3] Pedersen F., Butrymovich V., Kelbæk H., Wachtell K., Helqvist S., Kastrup J., Holmvang L., Clemmensen P., Engstrøm T., Grande P. (2014). J. Am. Coll. Cardiol..

[cit4] Terkelsen C., Christiansen E., Sørensen J., Kristensen S., Lassen J. F., Thuesen L., Andersen H. R., Vach W., Nielsen T. T. (2009). Heart.

[cit5] J. J. W. Group (2008). Circ. J..

[cit6] C. f. D. C. a. Prevention , Coronary Artery Disease (CAD), https://www.cdc.gov/heart-disease/about/coronary-artery-disease.html?CDC_AAref_Val=https://www.cdc.gov/heartdisease/coronary_ad.htm, (accessed 7/4, 2024)

[cit7] Acharya U. R., Hagiwara Y., Koh J. E. W., Oh S. L., Tan J. H., Adam M., San Tan R. (2018). Biocybern. Biomed. Eng..

[cit8] Asatryan B., Vaisnora L., Manavifar N. (2019). J. Am. Coll. Cardiol..

[cit9] Sabbagh C., Rahi M., Baz M., Haddad F., Helwe O., Aoun N., Ibrahim T., Abdo L. (2014). Am. J. Emerg. Med..

[cit10] Garg P., Morris P., Fazlanie A. L., Vijayan S., Dancso B., Dastidar A. G., Plein S., Mueller C., Haaf P. (2017). Intern. Emerg. Med..

[cit11] Tveit S. H., Myhre P. L., Hanssen T. A., Forsdahl S. H., Iqbal A., Omland T., Schirmer H. (2022). Sci. Rep..

[cit12] Dohi T., Maehara A., Brener S. J., Généreux P., Gershlick A. H., Mehran R., Gibson C. M., Mintz G. S., Stone G. W. (2015). Am. J. Cardiol..

[cit13] Chen Z., Li C., Lin K., Zhang Q., Chen Y., Rao L. (2018). Anatolian J. Cardiol..

[cit14] Summers S. M., Long B., April M. D., Koyfman A., Hunter C. J. (2018). Am. J. Emerg. Med..

[cit15] Wereski R., Kimenai D. M., Taggart C., Doudesis D., Lee K. K., Lowry M. T., Bularga A., Lowe D. J., Fujisawa T., Apple F. S. (2021). Circulation.

[cit16] Jaffe A. S., Ravkilde J., Roberts R., Naslund U., Apple F. S., Galvani M., Katus H. (2000). Circulation.

[cit17] Patibandla S. , Gupta K. and Alsayouri K., Cardiac enzymes, StatPearls Publishing, Treasure Island (FL), 2019 31424800

[cit18] Tanase D. M., Gosav E. M., Ouatu A., Badescu M. C., Dima N., Ganceanu-Rusu A. R., Popescu D., Floria M., Rezus E., Rezus C. (2021). Life.

[cit19] Kastrati A., Coughlan J. J., Ndrepepa G. (2021). J. Am. Coll. Cardiol..

[cit20] Schömig A., Ndrepepa G., Mehilli J., Schwaiger M., Schühlen H., Nekolla S., Pache J. r., Martinoff S., Bollwein H., Kastrati A. (2003). Circulation.

[cit21] Schulte C., Zeller T. (2015). Cardiovasc. Diagn. Ther..

[cit22] Jakob P., Kacprowski T., Briand-Schumacher S., Heg D., Klingenberg R., Stähli B. E., Jaguszewski M., Rodondi N., Nanchen D., Räber L. (2017). Eur. Heart J..

[cit23] Kim J. S., Pak K., Goh T. S., Jeong D. C., Han M.-E., Kim J., Oh S.-O., Kim C. D., Kim Y. H. (2018). Yonsei Med. J..

[cit24] Ghafouri-Fard S., Gholipour M., Taheri M. (2021). Front. Cardiovasc. Med..

[cit25] Melak T., Baynes H. W. (2019). eJIFCC.

[cit26] Dufourd T., Robil N., Mallet D., Carcenac C., Boulet S., Brishoual S., Rabois E., Houeto J.-L., de la Grange P., Carnicella S. (2019). Biol. Methods Protoc..

[cit27] Onuoha C. P., Ipe J., Simpson E., Liu Y., Skaar T. C., Kreutz R. P. (2022). Clin. Transl. Sci..

[cit28] Ren X., Ellis B. W., Ronan G., Blood S. R., DeShetler C., Senapati S., March K. L., Handberg E., Anderson D., Pepine C., Chang H.-C., Zorlutuna P. (2021). Lab Chip.

[cit29] Kumar S., Maniya N., Wang C., Senapati S., Chang H.-C. (2023). Nat. Commun..

[cit30] Ramshani Z., Zhang C., Richards K., Chen L., Xu G., Stiles B. L., Hill R., Senapati S., Go D. B., Chang H.-C. (2019). Commun. Biol..

[cit31] Taller D., Richards K., Slouka Z., Senapati S., Hill R., Go D. B., Chang H.-C. (2015). Lab Chip.

[cit32] Ramshani Z., Fan F., Wei A., Romanello-Giroud-Joaquim M., Gil C.-H., George M., Yoder M. C., Hanjaya-Putra D., Senapati S., Chang H.-C. (2021). Talanta.

[cit33] Maniya N. H., Kumar S., Franklin J. L., Higginbotham J. N., Scott A. M., Gan H. K., Coffey R. J., Senapati S., Chang H.-C. (2024). Commun. Biol..

[cit34] Tibshirani R. (1996). J. R. Stat. Soc. Ser. B Stat. Method..

[cit35] Fan J., Li R. (2001). J. Am. Stat. Assoc..

[cit36] Kanuri S. H., Ipe J., Kassab K., Gao H., Liu Y., Skaar T. C., Kreutz R. P. (2018). Atherosclerosis.

[cit37] Abdallah H. Y., Hassan R., Fareed A., Abdelgawad M., Mostafa S. A., Mohammed E. A.-M. (2022). BMC Cardiovasc. Disord..

[cit38] Witten A., Martens L., Schäfer A.-C., Troidl C., Pankuweit S., Vlacil A.-K., Oberoi R., Schieffer B., Grote K., Stoll M. (2022). Sci. Rep..

[cit39] Horváth M., Horváthová V., Hájek P., Štěchovský C., Honěk J., Šenolt L., Veselka J. (2020). Sci. Rep..

[cit40] Zhong Z., Hou J., Zhang Q., Zhong W., Li B., Li C., Liu Z., Yang M., Zhao P. (2018). Medicine.

[cit41] Xia K., Zhang Y., Sun D. (2020). Int. J. Mol. Med..

[cit42] Li W., Wang J., Hao W., Yu C. (2021). Biosci. Rep..

[cit43] Li W., Jin S., Hao J., Shi Y., Li W., Jiang L. (2021). Can. J. Physiol. Pharmacol..

[cit44] Lin B., Feng D., Xu J. (2019). Cell Biosci..

[cit45] Feng X., Lu J., Wu Y., Xu H. (2022). J. Physiol. Sci..

[cit46] Nabiałek E., Wańha W., Kula D., Jadczyk T., Krajewska M., Kowalówka A., Dworowy S., Hrycek E., Włudarczyk W., Parma Z. (2013). Minerva Cardioangiol..

[cit47] Su Q., Lv X., Ye Z., Sun Y., Kong B., Qin Z., Li L. (2019). Cell Death Dis..

[cit48] Rani A., Barter J., Kumar A., Stortz J. A., Hollen M., Nacionales D., Moldawer L. L., Efron P. A., Foster T. C. (2022). Aging.

[cit49] Karere G. M., Cox L. A., Bishop A. C., South A. M., Shaltout H. A., Mercado-Deane M.-G., Cuda S. (2021). J. Pediatr..

[cit50] Marconi L., Dabestani S., Lam T. B., Hofmann F., Stewart F., Norrie J., Bex A., Bensalah K., Canfield S. E., Hora M. (2016). Eur. Urol..

[cit51] Konoshenko M. Y., Lekchnov E. A., Vlassov A. V., Laktionov P. P. (2018). BioMed
Res. Int..

[cit52] Madsen L. H., Christensen G., Lund T., Serebruany V. L., Granger C. B., Hoen I., Grieg Z., Alexander J. H., Jaffe A. S., Van Eyk J. E. (2006). Circ. Res..

[cit53] Reichlin T., Hochholzer W., Bassetti S., Steuer S., Stelzig C., Hartwiger S., Biedert S., Schaub N., Buerge C., Potocki M. (2009). N. Engl. J. Med..

[cit54] Arshed S., Luo H. X., Zafar S., Regeti K., Malik N., Alam M., Yousif A. (2015). J. Clin. Med. Res..

[cit55] Wang B., Li Y., Hao X., Yang J., Han X., Li H., Li T., Wang D., Teng Y., Ma L. (2021). Front. Genet..

