# Supplementary material for: Towards real-time myocardial infarction diagnosis: a convergence of machine learning and ion-exchange membrane technologies leveraging miRNA signatures
Source: Lab Chip. 2024 Oct 4;24(23):5203–14. doi: 10.1039/d4lc00640b (PMC11484500; doi:10.1039/d4lc00640b)
Supplement: LC-024-D4LC00640B-s001 [file LC-024-D4LC00640B-s001.pdf]

Supplementary Information (SI) for Lab on a Chip.
This journal is © The Royal Society of Chemistry 2024

Supplementary Information

Towards Real-Time Myocardial Infarction Diagnosis: A Convergence of Machine Learning and Ion-Exchange Membrane Technologies Leveraging miRNA Signatures

Xiang Ren[†**], Ruyu Zhou[‡], George Ronan[†], S. Gulberk Ozcebe[†], Jiaying Ji[†], Satyajyoti Senapati[§], Keith L. March[^], Eileen Handberg[^], David Anderson[^], Carl J. Pepine[^], Hsueh-Chia Chang[§], Fang Liu[‡], Pinar Zorlutuna[†*]

† Department of Aerospace and Mechanical Engineering, University of Notre Dame, Notre Dame, IN 46556

**Current affiliation: Department of Microelectronics, Tianjin University, Tianjin, 300072, China

‡ Department of Applied and Computational Mathematics and Statistics, University of Notre Dame, Notre Dame, IN 46556

§ Department of Chemical and Biomolecular Engineering, University of Notre Dame, Notre Dame, IN 46556

^ Division of Cardiology, Department of Medicine in the College of Medicine, University of Florida, Gainesville, FL 32611

*Corresponding author: pinar.zorlutuna.1@nd.edu; Phone: (574)631-8543

1. Clinical samples

Blood was collected via standard venous puncture from healthy reference subjects (NCAD), patients diagnosed with CAD, and patients diagnosed with STEMI with an observed obstruction (STEMI-pre) and following reperfusion (STEMI-PCI) into tubes containing ethylenediaminetetraacetic acid (EDTA). After collection, plasma was isolated by centrifuging at 1000×g for 5 min and plasma was transferred to RNA free tubes and stored at -80°C and shipped to the University of Notre Dame. Once the samples arrived at Notre Dame, they were thawed on ice, aliquoted and stored at -80°C until experiments. The protocol was approved by University of Florida IRB (#201901232) and all subjects provided written informed consent.

2. Oligoprobes and calibration miRNAs

Table 1. Oligoprobes and the calibration miRNA.

miRNA name	Oligoprobes and molecular weight	Calibration and molecular weight
miR-1-1	5'-/5AmMC12/ATA CAT ACT TCT TTA CAT TCC A-3', MW=6866.7 g/mol	5'-TGG AAT GTA AAG AAG TAT GTA T-3', MW=6861.5 g/mol
miR-208b	5'-/5AmMC12/ACA AAC CTT TTG TTG GTC TTA T-3', MW=6913.7 g/mol	5'-ATA AGA CGA ACA AAA GGT TTG T-3', MW=6815.5 g/mol
miR-499-5p	5'-/5AmMC12/AAA CAT CAC TGC AAG TCT TAA-3', MW=6645.6 g/mol	5'-TTA AGA CTT GCA GTG ATG TTT-3', MW=6466.3 g/mol
miR-200b-3p	5'-/5AmMC12/TCA TCA TTA CCA GGC AGT ATT A-3', MW=6956.7 g/mol	5'-TAA TAC TGC CTG GTA ATG ATG A-3', MW=6773.5 g/mol
miR-543	5'-/5AmMC12/AAG AAG TGC ACC GCG AAT GTT T-3', MW=7046.8 g/mol	5'-AAA CAT TCG CGG TGC ACT TCT T-3', MW=6685.4 g/mol
miR-331-3p	5'-/5AmMC12/TTC TAG GAT AGG CCC AGG GGC-3', MW=6750.6 g/mol	5'-GCC CCT GGG CCT ATC CTA GAA-3', MW=6367.2 g/mol
miR-3605-5p	5'-/5AmMC12/GGC TTC CTT GCT ATC CAT CCT CA-3', MW=7164.8 g/mol	5'-TGA GGA TGG ATA GCA AGG AAG CC-3', MW=7186.7 g/mol
miR-301a-3p	5'-/5AmMC12/GCT TTG ACA ATA CTA TTG CAC TG-3', MW=7276.9 g/mol	5'-CAG TGC AAT AGT ATT GTC AAA GC-3', MW=7071.7 g/mol
miR-18a-5p	5'-/5AmMC12/CTA TCT GCA CTA GAT GCA CCT TA-3', MW=7221.9 g/mol	5'-TAA GGT GCA TCT AGT GCA GAT AG-3', MW=7127.7 g/mol
miR-423-5p	5'-/5AmMC12/AAA GTC TCG CTC TCT GCC CCT CA-3', MW=7158.8 g/mol	5'-TGA GGG GCA GAG AGC GAG ACT TT-3', MW=7193.7 g/mol
miR-142-5p	5'-/5AmMC12/AGT AGT GCT TTC TAC TTT ATG-3', MW=6680.5 g/mol	5'-CAT AAA GTA GAA AGC ACT ACT-3', MW=6431.3 g/mol
miR-132-3p	5'-/5AmMC12/CGA CCA TGG CTG TAG ACT GTT A-3', MW=7013.7 g/mol	5'-TAA CAG TCT ACA GCC ATG GTC G-3', MW=6719.4 g/mol

3. Integration board

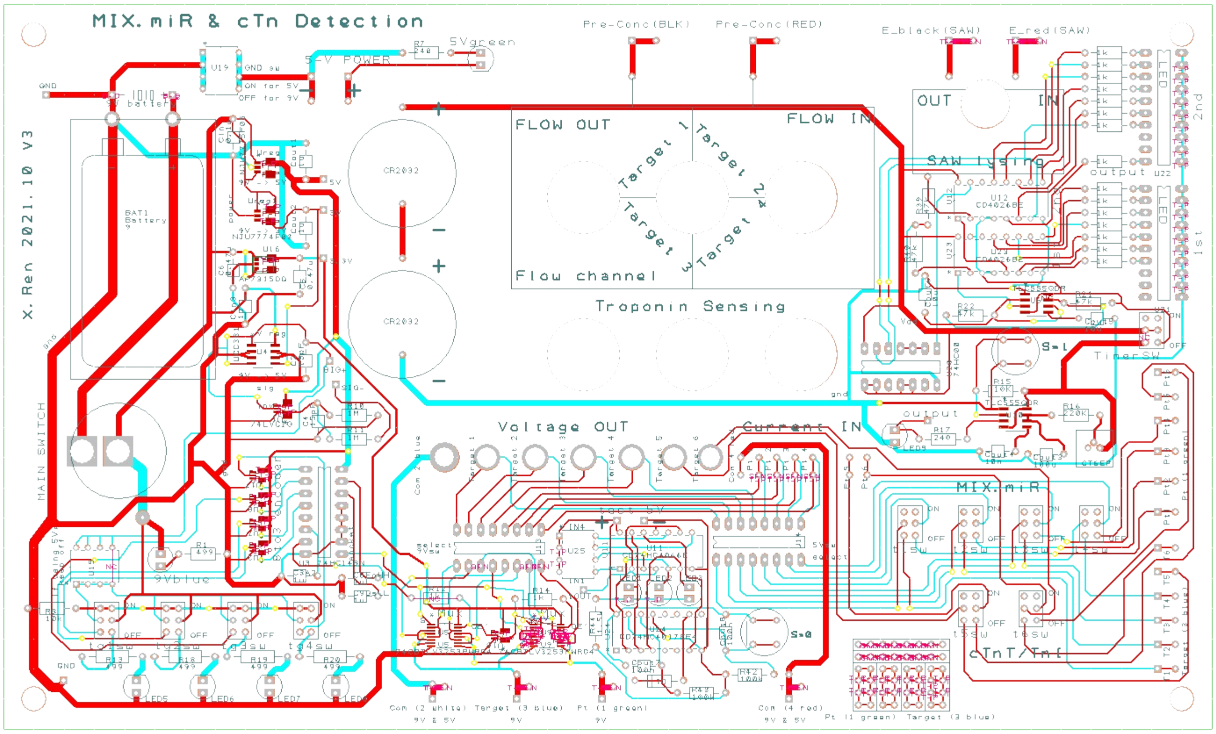

Figure S1. The layout design of the integration board.

4. Additional information of the selected patient samples

Random samples

	Age	Gender	Diabetes	Onset Hours (pre/post)	Troponin I (pg/mL)
**NCAD**	47	F	No		
	23	F	No		
	38	M	No		
**CAD**	57	M	Unknown		
	73	M	No		
	49	F	Yes		
**MI-Pre/PCI**	55	M	No	2.73→3.59	799
	65	F	No	53.98→54.86	NA
	80	F	Yes	98.15→98.7	3966
	45	M	No	5.45→5.9	868
	58	F	No	97.55→98.18	<6
	50	M	No	32.72→33.24	49

Matched samples

	Age	Gender	Diabetes	Onset Hours (pre/post)	Troponin I (pg/mL)
**NCAD**	63	F	No		
	65	F	No		
	63	M	No		
	63	M	No		
	67	F	No		
	67	M	No		
**CAD**	63	M	No		
	65	M	No		
	62	F	No		
	65	F	No		
	61	F	No		
	66	M	No		
**MI-Pre/PCI**	58	F	No	2.57→3.29	8
	50	M	No	1.65→2.48	27000
	67	M	No	5.55→6.05	2170
	64	F	No	1.25→2.25	6
	73	M	No	2.45→3.53	30
	66	M	No	1.27→1.80	11829

5. MIX.miR binding affinity of different miRNAs

miRNA binding affinity

$$\Delta G = RT \cdot ln\left(\frac{[DNA \cdot Primer]}{[DNA][Primer]}\right)$$

miRNA	Δ G (kcal/mol)
miR-1	22.9
miR-208b	25.5
miR-499	23.9
miR-200b	25.1
miR-543	29.8
miR-331	28.9
miR-3605	30.2
miR-301a	27
miR-18a	27.7
miR-423	32.5
miR-142	22.6
miR-132	28.5
